# Complex antiarrhythmic effects of empagliflozin in non-ischemic HFrEF cardiomyocytes

**DOI:** 10.3389/fphar.2026.1955044

**Published:** 2026-10-20

**Authors:** Bence Hegyi, Donald M. Bers

**Affiliations:** Department of Pharmacology, University of California, Davis, CA, United States

**Keywords:** cardiac arrhythmia, heart failure, ion channels, late sodium current, SGLT2 inhibitors, ventricular action potential

## Abstract

**Introduction:**

Sodium-glucose cotransporter-2 (SGLT2) inhibitors improve clinical outcomes in heart failure with reduced ejection fraction (HFrEF) and may reduce sudden cardiac death; however, the underlying antiarrhythmic mechanisms remain incompletely understood.

**Methods:**

We investigated the cellular electrophysiological effects of the SGLT2 inhibitor empagliflozin in isolated cardiomyocytes from two non-ischemic, non-diabetic HFrEF models (rabbit and mouse).

**Results:**

Ventricular cardiomyocytes from both HFrEF models exhibited prolonged action potential duration (APD), increased short-term APD variability (STV), and greater susceptibility to APD alternans and delayed afterdepolarizations (DADs). Intracellular Ca2+ buffering with EGTA prevented DADs, attenuated APD prolongation, and reduced repolarization instability. HFrEF cardiomyocytes also showed enhanced late Na+ current (INa,L) and reduced inward rectifier K+ current (IK1). Cell pretreatment with empagliflozin (1 μmol/L, 3 h), but not acute application (3 min), attenuated proarrhythmogenic AP changes, reduced INa,L, and increased IK1 in both species. Empagliflozin had no significant electrophysiological effects in healthy control cardiomyocytes. Selective inhibition of Na+/H+ exchanger-1 (NHE-1) with cariporide (1 μmol/L, 3 h) failed to reproduce the antiarrhythmic effects of empagliflozin under our experimental conditions; however, our studies were performed with intracellular pH buffered to 7.2, where NHE-1 activity is low.

**Discussion:**

These results demonstrate that empagliflozin attenuates key proarrhythmic electrophysiological changes in non-ischemic HFrEF cardiomyocytes. The requirement for drug pretreatment supports the hypothesis that empagliflozin acts through modulation of ion channel functional expression and/or post-translational regulation rather than direct acute channel effects in non-ischemic HFrEF cardiomyocytes.

## 1 Introduction

Empagliflozin, an inhibitor of sodium-glucose cotransporter-2 (SGLT2), significantly improves cardiovascular outcomes by reducing heart failure hospitalizations and cardiovascular death in patients with heart failure (HF) across all ejection fraction (EF) classes (Packer et al., 2020; Anker et al., 2021; Heidenreich et al., 2022). Empagliflozin is an antidiabetic drug that inhibits the reabsorption of glucose in kidney proximal tubules, thus attenuating hyperglycemia (Scheen, 2014). However, empagliflozin provides similar benefits in HF patients with and without diabetes mellitus (DM) (Packer et al., 2020). Notably, empagliflozin exerts direct effects on the heart despite SGLT2 expression being very low or absent in cardiomyocytes (Di Franco et al., 2017; Mustroph et al., 2018; Marfella et al., 2022). Preclinical studies have demonstrated that empagliflozin has multiple beneficial effects in *ex vivo* perfused hearts and isolated myocytes and was protective against HF even in SGLT2 knockout mice (Berger et al., 2024; Chen et al., 2024), indicating off-target effects (Bertero et al., 2018; Lopaschuk and Verma, 2020; Dyck et al., 2022; Hegyi and Bers, 2023). Emerging evidence suggests that empagliflozin may reduce cardiac arrhythmias in patients with DM and HF (Chen et al., 2020; Curtain et al., 2021; Fujiki et al., 2024). Its antiarrhythmic effects have been demonstrated in human induced pluripotent stem cell-derived cardiomyocytes (hiPSC-CMs) (Silva Dos Santos et al., 2023) and in animal models of ischemia (Azam et al., 2021), pharmacologically-induced long QT syndrome (Wolfes et al., 2024), DM (Kadosaka et al., 2023), and HF (Hegyi et al., 2022a; Hegyi et al., 2022b; Wen et al., 2024; Mira Hernandez et al., 2025; Mira Hernandez et al., 2026); however, the exact antiarrhythmic properties and molecular mechanisms of empagliflozin remain incompletely understood (Caballero et al., 2026).

Alterations in ionic currents and impaired Ca^2+^ handling play central roles in HF, contributing to both contractile dysfunction and arrhythmogenesis (Pogwizd et al., 2001; Bers et al., 2003; Landstrom et al., 2017; Hegyi et al., 2019b). Empagliflozin has been shown to modulate ionic fluxes in cardiomyocytes (Bertero et al., 2018; Dyck et al., 2022; Hegyi and Bers, 2023), including the cardiac Na^+^/H^+^ exchanger (NHE-1) (Baartscheer et al., 2017; Iborra-Egea et al., 2019; Trum et al., 2020; Chen et al., 2024), the late Na^+^ current (I_Na,L_) (Philippaert et al., 2021; Hegyi et al., 2022a; Hegyi et al., 2022b; Mira Hernandez et al., 2025), and the dysfunctional Ca^2+^ handling via suppression of chronically active Ca^2+^/calmodulin-dependent protein kinase II (CaMKII) in heart diseases (Mustroph et al., 2018; Mustroph et al., 2022; Hnatiuk et al., 2025). However, there are still some controversies about these being direct targets of empagliflozin (Chung et al., 2021; Murphy and Eisner, 2021; Hegyi and Bers, 2023). Importantly, empagliflozin pretreatment, but not acute application, attenuated the enhanced I_Na,L_ and CaMKII activity in failing myocytes in both HF with reduced EF (HFrEF) and in HF with preserved EF (HFpEF) (Mustroph et al., 2018; Hegyi et al., 2022b; Mira Hernandez et al., 2025), suggesting the likely involvement of cell signaling pathways and posttranslational regulatory mechanisms (PTMs). In line with this, empagliflozin reduced levels of reactive oxygen species (ROS) (Li et al., 2019) and intracellular *O*-linked β-N-acetylglucosamine (*O*-GlcNAc)-modified proteins (Kadosaka et al., 2023), both of which are key regulators of cardiac ion channels, Ca^2+^ handling, and CaMKII activity (Wagner et al., 2011; Hegyi et al., 2019a; Hegyi et al., 2021a).

In this study, we aimed to determine how empagliflozin modulates cardiomyocyte electrophysiological properties and arrhythmogenic mechanisms in non-ischemic HFrEF and to compare these effects with those of NHE-1 inhibitor cariporide (HOE-642). We have previously demonstrated that empagliflozin pretreatment reverses arrhythmogenic action potential (AP) remodeling in isolated cardiomyocytes from obese-diabetic HFpEF mice (Hegyi et al., 2022a; Hegyi et al., 2022b; Mira Hernandez et al., 2025). Based on these findings, we hypothesized that empagliflozin would similarly restore repolarization stability in HFrEF cardiomyocytes. Because hyperglycemia can further exacerbate arrhythmogenic remodeling and glucose lowering may provide additional electrophysiological benefits (Hegyi et al., 2019a; Hegyi et al., 2020a; Lopaschuk and Verma, 2020; Hegyi et al., 2021a; Hegyi et al., 2021b), we focused on non-diabetic HFrEF to isolate glucose-independent effects of empagliflozin. To enhance translational relevance, we used both murine and rabbit models of chronic non-diabetic, non-ischemic HFrEF, as rabbit cardiac electrophysiology more closely resembles that of humans (Morotti et al., 2021; Hornyik et al., 2022), whereas most prior cardiomyocyte-level studies of empagliflozin have been conducted in mice.

## 2 Materials and methods

### 2.1 Ethical approval

All animal procedures strictly followed the approved protocols of the Institutional Animal Care and Use Committee at University of California, Davis (#22805 and #23175) and were conducted in accordance with the US National Institutes of Health Guide for the Care and Use of Laboratory Animals (8^th^ edition, 2011).

### 2.2 Animal procedures

Animals were housed under controlled conditions of temperature, humidity, and a 12-h light/dark cycle, with standard chow and drinking water provided *ad libitum*.

HFrEF in male New Zealand white rabbits (3-month-old at study entry; Charles River Laboratories) was induced by mild aortic insufficiency using a cutting-balloon catheter, followed 4 weeks later by abdominal aortic banding guided by a 3.5F catheter inserted through the femoral artery, as previously described (Pogwizd et al., 2001; Hegyi et al., 2018a; Hegyi et al., 2019b). Chronic HF progression was monitored longitudinally by transthoracic echocardiography and regular assessments for clinical signs of HF by veterinarians and trained staff. Cardiomyocytes were isolated when HFrEF developed with left ventricular end-systolic dimension >1.4 cm and EF<35%, typically at 2–4 years of age. Data here was obtained from 11 HFrEF and 13 age-matched healthy control rabbits.

HFrEF in C57BL/6J wild-type mice (male, 8-week-old at study entry, the Jackson Laboratory, strain#: 000664) was induced by pressure overload via transverse aortic constriction (TAC) as previously described (Bossuyt et al., 2022). Briefly, mice were anesthetized with 2%–4% isoflurane in 100% O_2_, and a left thoracotomy was performed at the 2^nd^ intercostal space. The transverse aorta was ligated with a suture tied against a 28-gauge needle. Successful aortic constriction was confirmed by measurement of the pressure gradient and by observation of reduced left carotid artery perfusion. Severe HFrEF, defined by EF<25%, typically developed within 8 weeks after TAC, at which time cardiomyocytes were isolated. Data here was obtained from 10 TAC and 10 sham-operated control mice

All HFrEF animals exhibited significant cardiac hypertrophy, pulmonary edema, and abdominal ascites, consistent with findings from our previous studies using these HFrEF models (Hegyi et al., 2018a; Hegyi et al., 2019b; Bossuyt et al., 2022). Only male mice and rabbits were used because young adult females are relatively protected against HFrEF in both species.

For terminal procedures, animals were injected with heparin (400 U/kg, IV) and maintained under anesthesia with 3%–5% isoflurane inhalation. Animals were euthanized by surgical excision of the heart while under deep anesthesia. After confirmation of absent pain reflexes, the hearts were excised and rinsed in cold, nominally Ca^2+^-free minimal essential medium. The aorta was cannulated, and the heart was retrogradely perfused using a constant-flow Langendorff apparatus at 37 °C with Ca^2+^-free normal Tyrode’s solution, gassed with 100% O_2_. The heart was then perfused with collagenase (type 2, Worthington; 320 U/mg specific collagenase activity; Cat#: LS004177; Lot#: F7P17939B) and protease (type XIV, Sigma-Aldrich; 5.9 U/mg specific protease activity; Cat#: P5147; Batch#: 0000435293) in Tyrode’s solution (with 10 μmol/L free [Ca^2+^]) for to enzymatically isolate cardiomyocytes, as previously described (Hegyi et al., 2021b). For rabbits, 0.69 g/L collagenase and 6.2 mg/L protease XIV were perfused for 25–35 min. For mice, 1.38 g/L collagenase and 6.4 mg/L protease XIV were perfused for 10–18 min. Ventricular cardiomyocytes were collected from the midmyocardial region of the left ventricular free wall. Following digestion, cardiomyocytes were gently triturated with a pipette, filtered through a 200-μm nylon mesh, and allowed to sediment for 10 min. This sedimentation step was repeated three times with progressively increasing [Ca^2+^] (0.125, 0.25, and 0.5 mmol/L). Finally, ventricular cardiomyocytes were kept in Tyrode’s solution at room temperature until use.

### 2.3 Cardiomyocyte electrophysiology

APs and ionic currents were recorded in ventricular cardiomyocytes using the patch-clamp technique at 37 °C (Mira Hernandez et al., 2025). Following isolation, cardiomyocytes were randomly allocated to treatment groups and incubated for 3 h with vehicle (0.1% DMSO), empagliflozin (1 μmol/L in 0.1% DMSO), or cariporide (1 μmol/L in 0.1% DMSO). An empagliflozin concentration of 1 μmol/L was selected based on its approximate relevance to plasma concentrations achievable *in vivo* (C_max_) following therapeutic dosing in human patients (Scheen, 2014). Aliquots were randomly selected for electrophysiological recordings, and recordings were performed within 6 h of cell isolation to minimize potential confounding effects related to the time elapsed between isolation and measurement. Cardiomyocytes were placed in a temperature-controlled perfusion chamber (Warner) mounted on a Leica DMI3000 B inverted microscope and continuously superfused at 2 mL/min with Tyrode’s solution containing (mmol/L): 140 NaCl, 4 KCl, 1.8 CaCl_2_, 1 MgCl_2_, 5 HEPES, 5 Na-HEPES, 5.5 glucose, pH = 7.40 (with NaOH). Patch electrodes were fabricated from borosilicate glass (WPI) and had tip resistances of 2–4 MΩ. Recordings were performed using an Axopatch 200B amplifier (Axon), and the signals were digitized at 50 kHz by a Digidata 1322A/D converter (Axon) under software control (pClamp10.4). Rod-shaped cardiomyocytes with clear cytoplasm, regular striations, and well-defined, linear cell edges, were selected for electrophysiological experiments. In the ruptured-patch configuration, series resistance was typically 3–5 MΩ and it was compensated by ≥ 80%. Experiments were excluded if the series resistance was excessively high or increased by >20% during the recordings.

For AP and I_K1_ measurements, pipette solution contained (mmol/L): K-aspartate 100, KCl 30, NaCl 8, Mg-ATP 5, phosphocreatine dipotassium salt 10, HEPES 10, EGTA 0.01, cAMP 0.002, and calmodulin 0.0001; pH = 7.20 (with HCl). Using this internal solution, the intracellular Ca^2+^ transients and cardiomyocyte contraction were preserved (Hegyi et al., 2018b; Hegyi et al., 2021d). To further assess the Ca^2+^-dependence of proarrhythmic mechanisms, APs were also recorded using an internal solution in which EGTA was increased to 10 mmol/L with free [Ca^2+^] = 100 nmol/L. APs were recorded in whole-cell current-clamp conditions where cells were stimulated using supra-threshold, 2-ms-long depolarizing pulses delivered via the patch pipette. Fifty consecutive APs were analyzed to estimate short-term variability (STV) of APD_90_ according to the following formula: STV = Σ(│APD_n+1_−APD_n_│)/[(n_beats_−1) × √2], where APD_n_ and APD_n+1_ indicate the *n*th and (n+1)^th^ APDs, and n_beats_ denotes the total number of consecutive beats analyzed (Szentandrassy et al., 2015). Spontaneous diastolic activities were recorded for 1 min following cessation of 1-min tachypacing (5 Hz in rabbits and 10 Hz in mice). Delayed afterdepolarizations (DADs) were defined as depolarizations>2 mV (>4-fold the electrical noise amplitude, allowing unambiguous automated detection) with the peak depolarization occurring within 0.5 s of DAD onset. Spontaneous APs (sAPs) were defined as depolarizations showing overshoot with a rapid upstroke phase. I_K1_ was measured at the end of 100-ms test pulses to −140 mV and −40 mV from a holding potential of −80 mV. The I_K1_ measured under these conditions was inhibited by BaCl_2_ (300 μmol/L) at the end of the experiments, confirming its identity.

For I_Na,L_ measurements, pipette solution contained (mmol/L): 110 CsCl, 20 tetraethylammonium chloride, 5 MgATP, 10 HEPES, 5 phosphocreatine-Na_2_, 0.0001 calmodulin, 10 EGTA, 4.1 CaCl_2_ (free [Ca^2+^] = 100 nmol/L); pH = 7.20 (with HCl). The bath solution contained (mmol/L): 140 NaCl, 4 CsCl, 1.8 CaCl_2_, 1 MgCl_2_, 5 HEPES, 5 Na-HEPES, 5.5 glucose, 5 4-aminopyridine, 0.01 nifedipine; pH = 7.40 (with NaOH). Although cytosolic [Na^+^] is expected to partially equilibrate with the pipette solution, submembrane [Na^+^] may remain distinct, particularly during repetitive stimulation (Hegyi et al., 2016; Skogestad et al., 2019). I_Na,L_ was measured at the end of a 500 ms depolarizing pulse to −40 mV from a holding potential of −120 mV (to maximize Na^+^ channel availability). I_Na,L_ was quantified as the tetrodotoxin (TTX, 10 μmol/L)-sensitive current amplitude normalized to membrane capacitance, determined using short (10 ms) hyperpolarizing pulses from −10 mV to −20 mV.

### 2.4 Statistics

Data are presented as Mean ± SD. Statistical significance of differences was determined using hierarchical (nested) ANOVA followed by appropriate *post hoc* tests in pairwise comparisons. GraphPad Prism 11 was used for data analysis. *P* < 0.05 was considered statistically significant.

## 3 Results

### 3.1 Empagliflozin pretreatment attenuates AP changes in murine and rabbit HFrEF cardiomyocytes

We tested the direct electrophysiological effects of empagliflozin on cardiomyocyte using two established non-ischemic, non-diabetic animal models of chronic HFrEF. In the first model, freshly isolated murine left ventricular myocytes from transverse aortic constriction (TAC)-induced HFrEF mice and sham controls were used (Figure 1A). In the second model, cardiomyocytes from rabbits with combined pressure- and volume-overload-induced chronic HFrEF and age-matched controls were used (Figure 1B). In both models, HFrEF cardiomyocytes exhibited marked prolongation of AP duration at 90% repolarization (APD_90_), significantly increased short-term variability of APD_90_ (STV), and reduced maximal upstroke velocity (dV/dt_max_) compared with healthy controls (Figure 1). Resting membrane potential (RMP) was significantly depolarized in HFrEF rabbits (Figure 1H) and showed a trend toward depolarization in TAC mice (Figure 1G). Acute perfusion (3 min) with empagliflozin (1 μmol/L) had no effect on any AP parameters in either HFrEF or control cardiomyocytes (Figure 1). In contrast, empagliflozin pretreatment (3 h) in murine and rabbit HFrEF cardiomyocytes markedly attenuated APD_90_ prolongation and reduced STV (Figures 1E,F). Empagliflozin pretreatment also improved RMP in HFrEF rabbits (Figure 1H) and increased dV/dt_max_ in TAC mice (Figure 1G). No effects of 3-h empagliflozin treatment were observed in control myocytes (Figure 1). Treatment with the NHE-1 inhibitor cariporide (1 μmol/L) for 3 h did not ameliorate electrophysiological alterations in HFrEF and had no effects in controls (Figure 1). Together, these data demonstrate that empagliflozin exerts beneficial effects on ventricular APs following 3 h of cell pretreatment, but not during acute application, and that these effects extend beyond NHE-1 inhibition.

**FIGURE 1:**
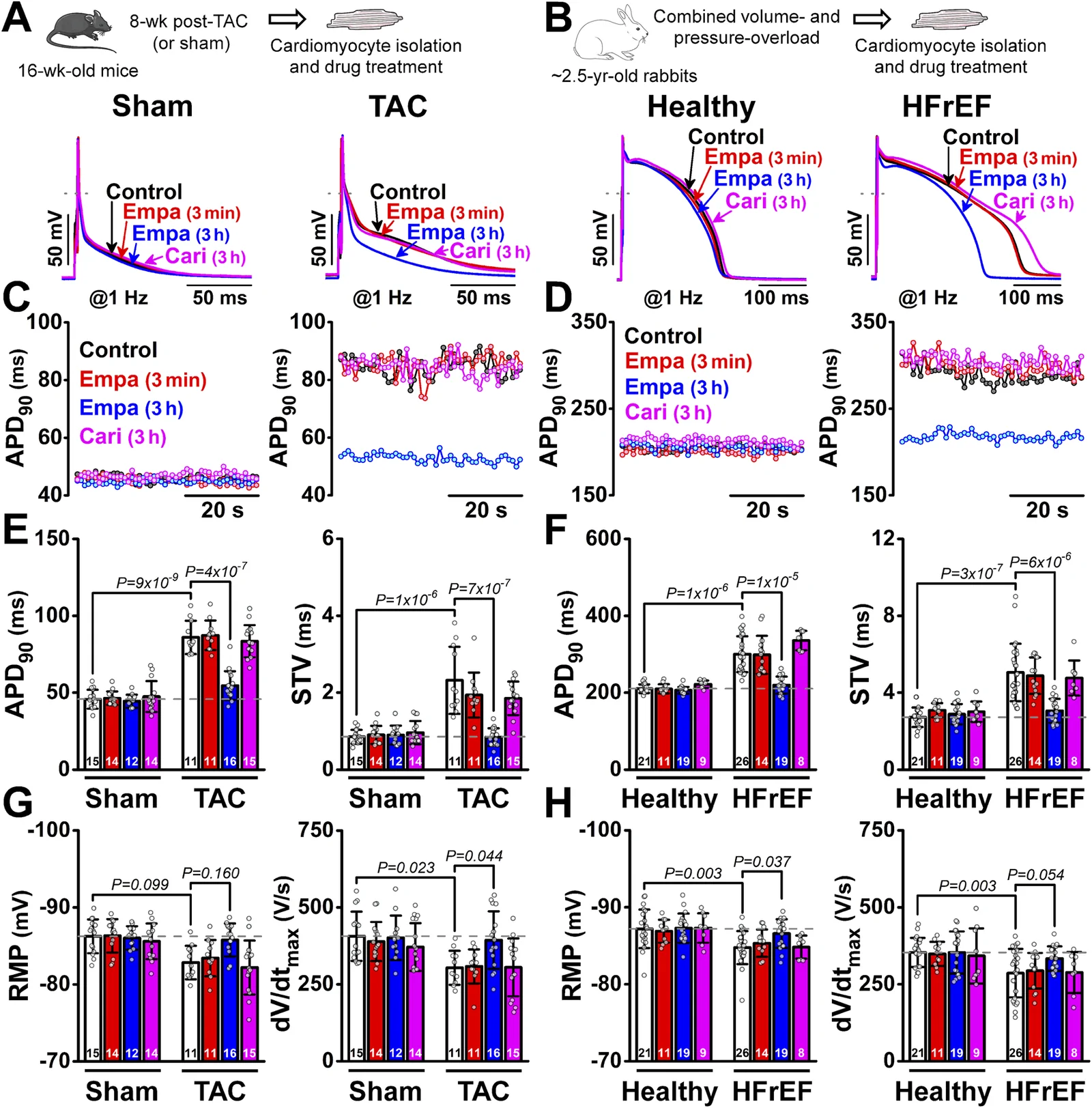
Empagliflozin pretreatment attenuates proarrhythmogenic AP changes in HFrEF murine and rabbit ventricular myocytes. **(A)** Representative action potentials (APs) in murine ventricular cardiomyocytes 8-week after transverse aortic constriction (TAC)-induced heart failure with reduced ejection fraction (HFrEF) or sham procedure. Cells were treated with vehicle (0.1% DMSO, 3 h), empagliflozin (3 min or 3 h), and cariporide (3 h). **(B)** Representative APs in rabbit ventricular cardiomyocytes in HFrEF induced by combined volume- and pressure-overload and in healthy age-matched controls. **(C,D)** Fifty consecutive APD_90_ values were used to calculate short-term variability of APD_90_ (STV) in mice **(C)** and rabbits **(D)**. **(E,F)** AP duration at 90% repolarization (APD_90_) and STV in murine TAC **(E)** and HFrEF rabbit **(F)** cardiomyocytes. **(G,H)** Resting membrane potential (RMP) and maximal upstroke velocity (dV/dt_max_) in murine **(G)** and rabbit **(H)** HFrEF cardiomyocytes. Empagliflozin pretreatment (3 h) attenuated AP changes in both HFrEF models, while acute empagliflozin treatment (3 min) or pretreatment with cariporide (3 h) had no effect on any AP parameters. Hierarchical (nested) ANOVA followed by Dunnett’s multiple comparisons test. N = 5 control and 5 HFrEF mice, and 7 control and 7 HFrEF rabbits. Individual cell data and number of cells in each treatment group are shown in the figure.

### 3.2 Empagliflozin pretreatment reduces alternans and DAD susceptibility in HFrEF cardiomyocytes

Prolonged AP repolarization, reduced dV/dt_max_, and increased STV observed in HFrEF cardiomyocytes are consistent with an arrhythmogenic substrate that was attenuated by empagliflozin pretreatment. We further tested the effects of empagliflozin on key cellular proarrhythmic mechanisms, including APD alternans and DADs (Weiss et al., 2011).

In TAC myocytes, significant APD_90_ alternans occurred at rapid steady-state pacing frequencies of ≥7 Hz, corresponding to the physiological heart rate range in mice (Figure 2A). In HFrEF rabbit myocytes, APD_90_ alternans threshold was reduced to 3 Hz compared to 5 Hz in control (Figure 2B). Empagliflozin pretreatment, but not acute application, increased the alternans threshold frequency and reduced the magnitude of APD_90_ alternans (defined as the difference between consecutive long and short beats) in both murine and rabbit HFrEF cardiomyocytes (Figures 2A–D). In TAC mice, APD_90_ alternans was observed in all cells (15/15) at 10 Hz pacing, whereas only 1/10 cells exhibited alternans following empagliflozin pretreatment, and no alternans was observed in control myocytes at 10 Hz pacing (Figure 2G). In HFrEF rabbits, empagliflozin pretreatment reduced APD_90_ alternans magnitude to control levels (Figure 2H).

**FIGURE 2:**
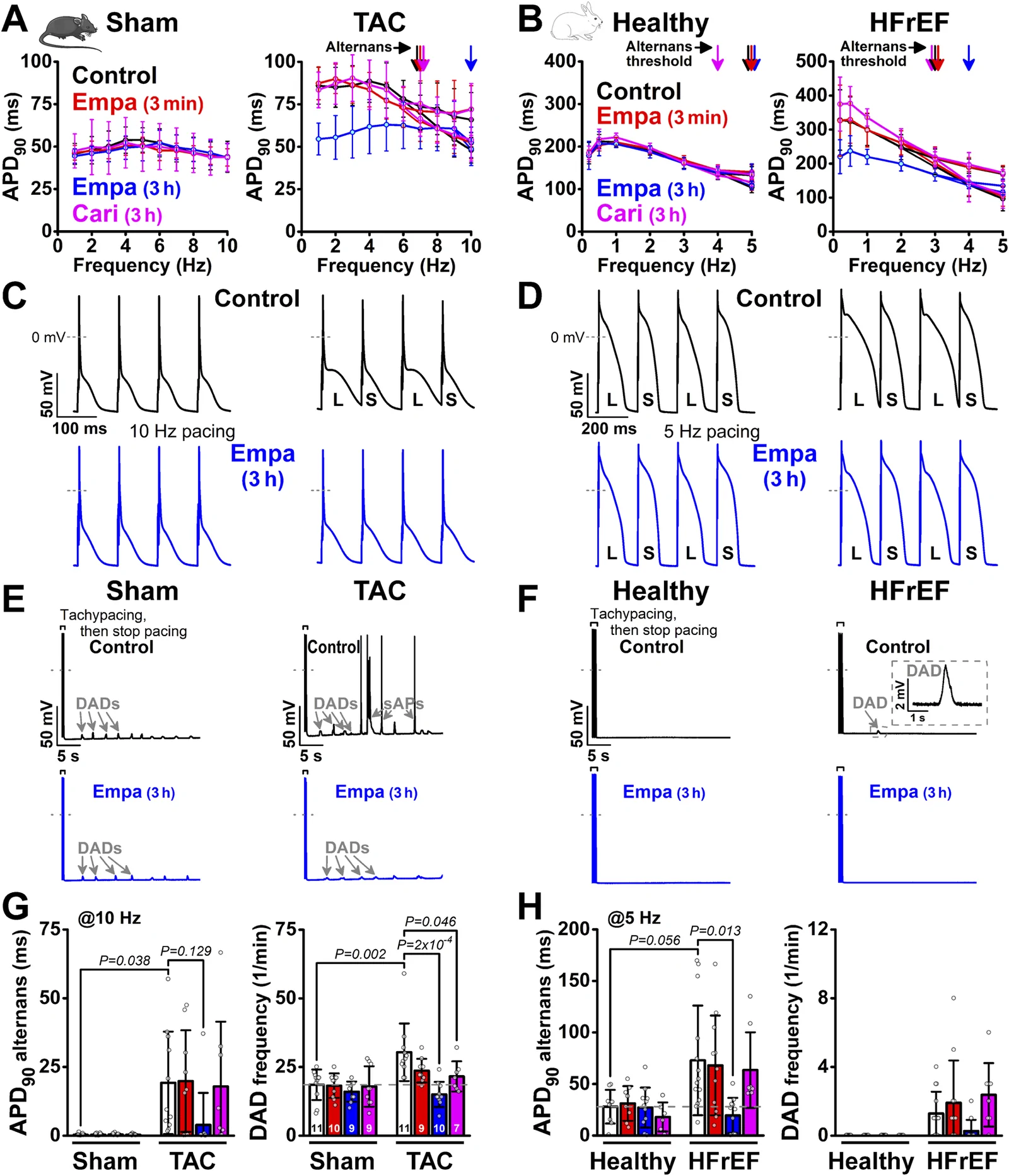
Empagliflozin pretreatment attenuates AP alternans and spontaneous diastolic activities in HFrEF ventricular myocytes. **(A,B)** Frequency-dependence of AP duration at 90% repolarization (APD_90_) in murine TAC and sham ventricular cardiomyocytes **(A)** and in rabbit HFrEF and healthy control ventricular cardiomyocytes **(B)**. **(C,D)** APD_90_ alternans at 10 Hz pacing rate in TAC mice **(C)** and at 5 Hz pacing rate in HFrEF rabbits **(D)**. **(E,F)** Spontaneous action potentials (sAPs) and delayed afterdepolarizations (DADs) following cassation of tachypacing in TAC murine myocytes **(E)** and in HFrEF rabbit myocytes **(F)**. **(G,H)** APD_90_ alternans magnitude and spontaneous diastolic activities (DAD + sAP) in murine **(G)** and rabbit **(H)** myocytes. Empagliflozin (3 h) pretreatment attenuated APD_90_ alternans and reduced DAD frequency. Acute empagliflozin treatment (3 min) or pretreatment with cariporide (3 h) had no effect on APD_90_ alternans and only reduced DADs in TAC mice but not in HFrEF rabbits. Hierarchical (nested) ANOVA followed by Dunnett’s multiple comparisons test. N = 5 control and 5 HFrEF mice, and 7 control and 7 HFrEF rabbits. Individual cell data and number of cells in each treatment group are shown in the figure.

Frequent DADs were observed following cessation of tachypacing in HFrEF cardiomyocytes (Figures 2E,F). In TAC mice, empagliflozin pretreatment significantly reduced DAD incidence and prevented spontaneous APs (Figures 2E,G). In HFrEF rabbits, DADs occurred in 10/15 cells compared with 2/12 cells following empagliflozin pretreatment (*P* = 0.015, Fisher’s exact test), resulting in a trend toward reduced overall DAD frequency (Figures 2F,H). Acute empagliflozin treatment (3 min) had no effect on APD_90_ alternans or DAD occurrence in either HFrEF model (Figure 2). Cariporide pretreatment did not affect APD_90_ alternans in either HFrEF model (Figure 2). However, cariporide slightly reduced DAD incidence in TAC myocytes but had no effect in HFrEF rabbit cardiomyocytes (Figures 2G,H). Together, these findings demonstrate that empagliflozin pretreatment, but not acute exposure, suppresses multiple cellular proarrhythmic mechanisms in HFrEF, with effects that extend beyond NHE-1 inhibition, supporting a time-dependent, pleiotropic antiarrhythmic action of empagliflozin.

### 3.3 Intracellular Ca^2+^ buffering mimics the antiarrhythmic effects of empagliflozin in HFrEF cardiomyocytes

Impaired intracellular Ca^2+^ handling, including increased Ca^2+^ leak through the ryanodine receptor (RyR), slowed sarcoplasmic reticulum (SR) Ca^2+^ reuptake, and enhanced Na^+^/Ca^2+^ exchanger (NCX) activity, has been reported in HFrEF and contributes to arrhythmogenesis (Pogwizd et al., 2001; Bers et al., 2003; Ai et al., 2005; Landstrom et al., 2017; Hegyi et al., 2019b). To assess the Ca^2+^-dependence of the observed proarrhythmic electrophysiological changes, HFrEF cardiomyocytes were studied using a pipette solution containing 10 mmol/L EGTA (estimated free [Ca^2+^] = 100 nmol/L). Intracellular Ca^2+^ buffering with EGTA attenuated APD_90_ prolongation and reduced STV in both TAC mouse (Figure 3A) and HFrEF rabbit cardiomyocytes (Figure 3B). In addition, EGTA prevented the development of APD_90_ alternans and abolished DADs in both HFrEF models (Figures 3A,B), indicating that these electrophysiological abnormalities are predominantly Ca^2+^-dependent. These findings are consistent with a Ca^2+^-mediated mechanism underlying arrhythmogenic remodeling in HFrEF and raise the possibility that empagliflozin may exert antiarrhythmic effects, at least in part, by improving intracellular Ca^2+^ signaling. Given the central role of CaMKII in translating aberrant Ca^2+^ signaling into electrophysiological dysfunction, CaMKII may represent an important downstream mediator of these Ca^2+^-dependent effects (Ai et al., 2005; Hegyi et al., 2019a; Hegyi et al., 2019b).

**FIGURE 3:**
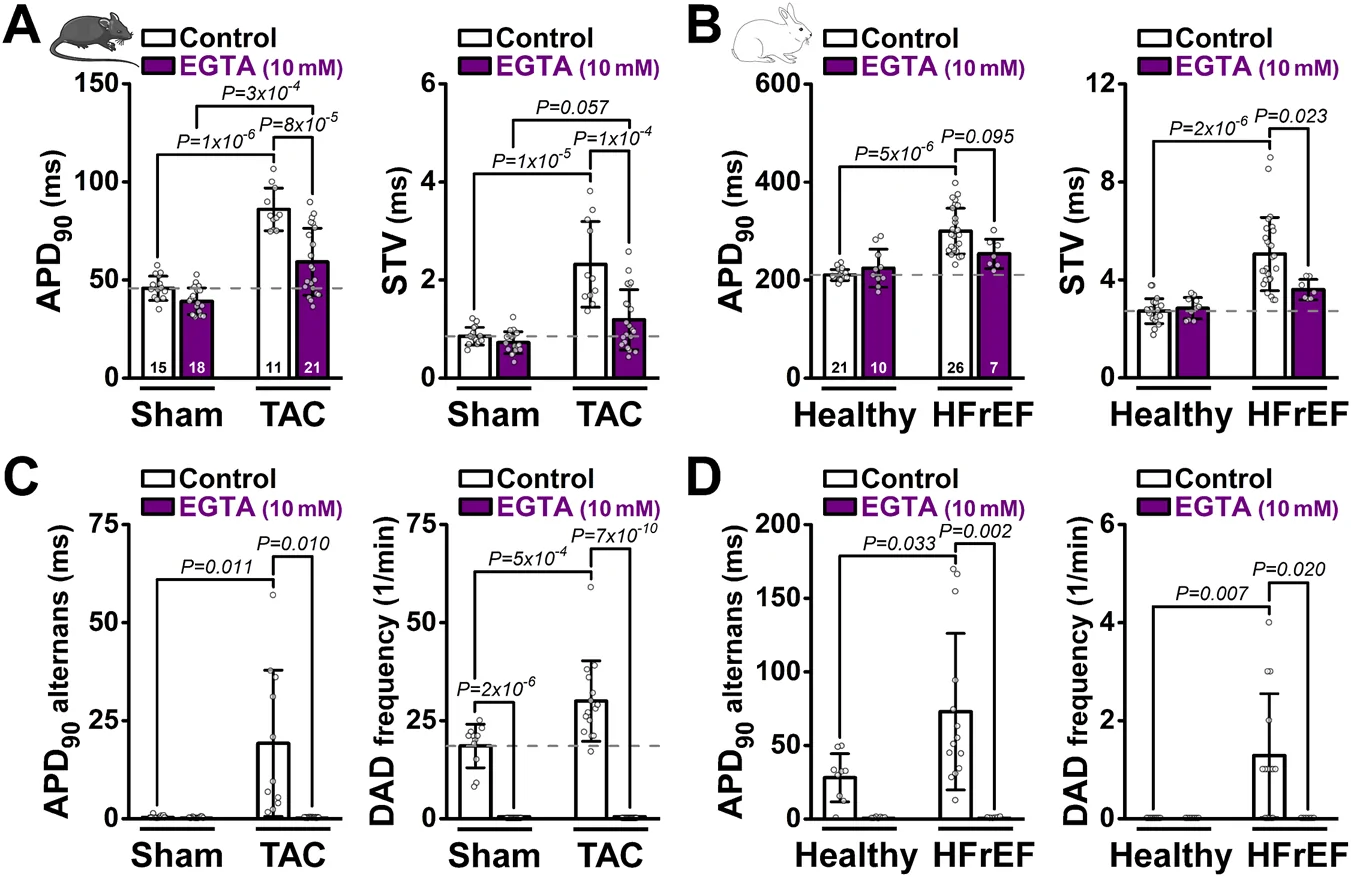
Arrhythmogenic AP changes are calcium-dependent in HFrEF cardiomyocytes. **(A,B)** Buffering of intracellular Ca^2+^ using 10 mmol/L EGTA attenuated APD_90_ prolongation and large short-term APD_90_ variability (STV) and prevented APD_90_ alternans and DADs both in TAC murine ventricular cardiomyocytes **(A)** and in HFrEF rabbit ventricular myocytes **(B)**. **(C,D)** Ca^2+^ buffering using EGTA prevented APD_90_ alternans and DADs in murine **(C)** and rabbit **(D)** myocytes. Nested ANOVA followed by Tukey’s multiple comparisons test. N = 5 control and 5 HFrEF mice, and 7 control and 7 HFrEF rabbits. Individual cell data and number of cells in each group are shown in the figure.

### 3.4 Empagliflozin ameliorates changes in I_Na,L_ and I_K1_ in HFrEF cardiomyocytes

The proarrhythmogenic AP changes observed in HFrEF are in line with alterations in key ionic currents. Enhanced I_Na,L_ and reduced I_K1_ have previously been shown to significantly contribute to impaired AP repolarization in HFrEF (Pogwizd et al., 2001; Marionneau et al., 2008; Hegyi et al., 2018a; Hegyi et al., 2019b; Bossuyt et al., 2022). Thus, we measured these ionic currents in voltage-clamped cardiomyocytes with or without empagliflozin pretreatment (Figures 4A,B,E,F). Because HFrEF myocytes were hypertrophic and exhibited increased membrane capacitance (Figures 4C,D), all ionic currents were normalized to capacitance, and current densities are reported.

**FIGURE 4:**
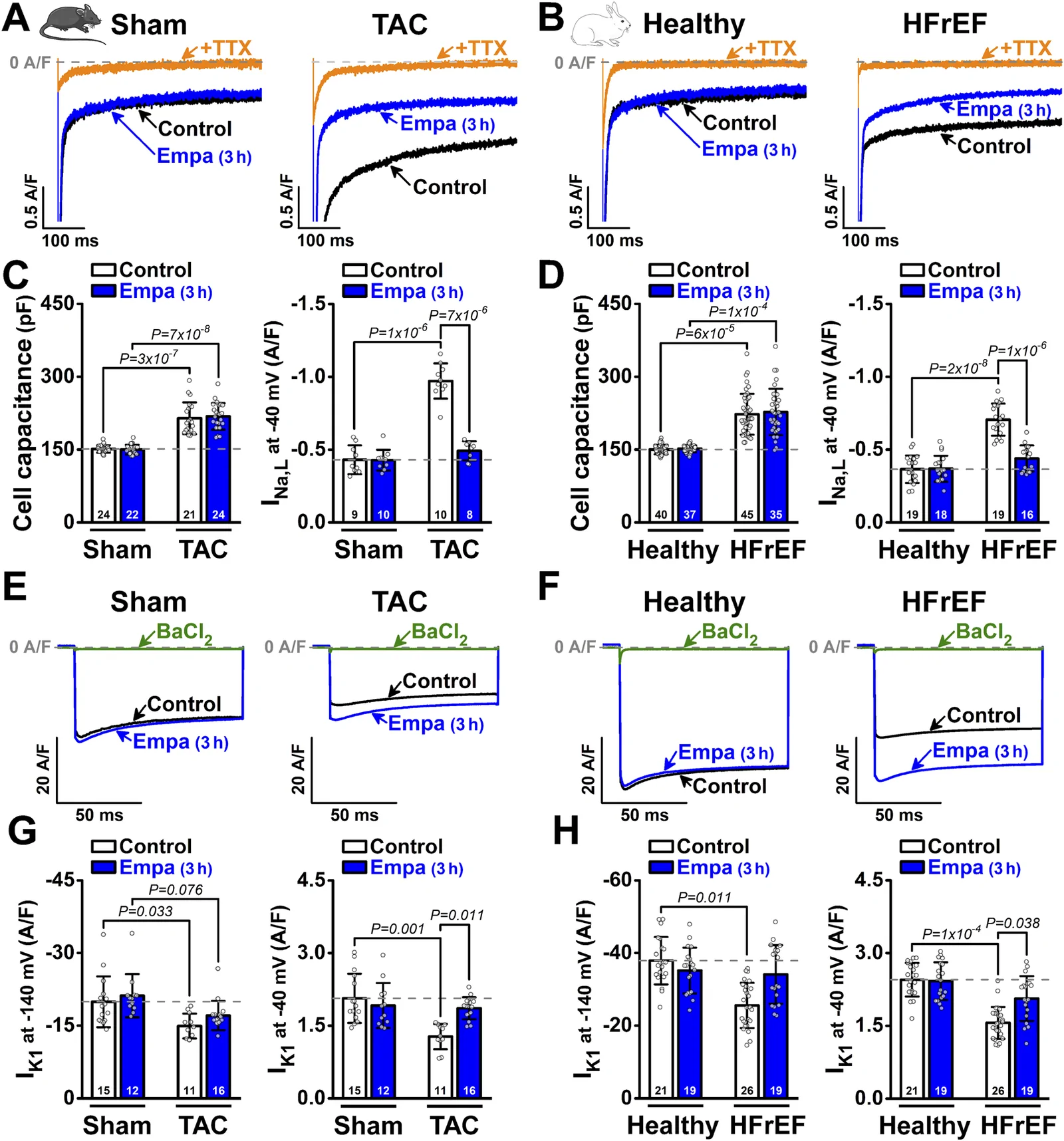
Empagliflozin pretreatment reverses both late sodium current enhancement and inward rectifier potassium current reduction in HFrEF cardiomyocytes. **(A,B)** Late Na^+^ current (I_Na,L_) traces at −40 mV in murine TAC and sham ventricular cardiomyocytes **(A)** and in rabbit HFrEF and control ventricular cardiomyocytes **(B)**. **(C,D)** I_Na,L_ density and cell capacitance in murine **(C)** and rabbit **(D)** myocytes. I_Na,L_ was markedly enhanced in both HFrEF models, and empagliflozin (3 h) reversed I_Na,L_ enhancement without affecting cell capacitance. Tetrodotoxin (TTX, 10 μmol/L) was applied at the end of the experiments to fully inhibit I_Na,L_. **(E,F)** Inward rectifier K^+^ current (I_K1_) traces at −140 mV in murine **(E)** and rabbit **(F)** myocytes. **(G,H)** I_K1_ was reduced in both the inward (−140 mV) and outward (−40 mV) directions, in both HFrEF models, and empagliflozin increased outward I_K1_. BaCl_2_ (300 μmol/L) was applied at the end of the experiments to inhibit I_K1_. Hierarchical (nested) ANOVA followed by Tukey’s multiple comparisons test. N = 4 animals for I_Na,L_ and N = 5 animals for I_K1_ for mice and N = 7 for rabbits. Individual cell data and number of cells in each group are shown in the figure.

I_Na,L_ was a tiny current in healthy control myocytes (Figures 4C,D), and I_Na,L_ density was slightly larger in murine vs. rabbit myocytes (−0.43 ± 0.10 A/F vs. −0.37 ± 0.09 A/F). However, I_Na,L_ density was markedly increased in HFrEF cardiomyocytes in both species (Figures 4C,D), with a greater increase in murine TAC myocytes (+126%) than in HFrEF rabbit myocytes (+93%). Empagliflozin pretreatment reversed I_Na,L_ enhancement in both HFrEF models and had no effect in control cardiomyocytes (Figures 4C,D).

I_K1_ in healthy control myocytes was greater in rabbit than in murine cardiomyocytes (Figures 4G,H), particularly in the inward direction (at −140 mV; −37.9 ± 6.5 A/F vs. −19.9 ± 5.2 A/F), and to a lesser extent in the outward direction (at −40 mV; 2.4 ± 0.3 A/F vs. 2.1 ± 0.5 A/F). I_K1_ was significantly reduced in HFrEF cardiomyocytes in both models with larger reductions in inward than outward I_K1_ (−39% vs. −25% in TAC mice, and −36% vs. −33% in HFrEF rabbits). Importantly, empagliflozin pretreatment significantly increased I_K1_ density in HFrEF cardiomyocytes and had no effect in control myocytes (Figures 4G,H). In HFrEF rabbits (Figure 4H), empagliflozin increased inward and outward I_K1_ to a similar extent (+33% and +32%, respectively). However, in TAC mice (Figure 4G), this increase was greater in the physiologically relevant outward than inward I_K1_ (+46% vs. +15%). These changes are consistent with the observed AP remodeling in HFrEF and demonstrate that empagliflozin ameliorates changes of both I_K1_ and I_Na,L_ functional expression in HFrEF cardiomyocytes.

## 4 Discussion

### 4.1 Empagliflozin modulates Na^+^ and Ca^2+^ homeostasis in heart failure

Empagliflozin is a key component of the guideline-directed medical therapy for HF (Heidenreich et al., 2022). As an SGLT2 inhibitor that reduces hyperglycemia, empagliflozin may provide additional benefits in patients with DM and HF (Lopaschuk and Verma, 2020). Importantly, empagliflozin improves clinical outcomes in HF beyond its antidiabetic actions (Cowie and Fisher, 2020), and it exerts direct functional benefits in failing human cardiomyocytes (Mustroph et al., 2018; Pabel et al., 2018). Empagliflozin may reduce cardiac arrhythmia susceptibility in patients with DM and HF (Chen et al., 2020; Curtain et al., 2021; Fujiki et al., 2024). Consistent with these clinical observations, empagliflozin exerts antiarrhythmic effects in hiPSC-CMs (Silva Dos Santos et al., 2023) and in diverse preclinical models, including ischemia (Azam et al., 2021), long QT syndrome (Wolfes et al., 2024), DM (Kadosaka et al., 2023), and HF (Hegyi et al., 2022a; Hegyi et al., 2022b; Wen et al., 2024; Mira Hernandez et al., 2025). Here, in HFrEF cardiomyocytes, we report that empagliflozin reversed APD prolongation and STV (Figure 1), sensitive markers for repolarization instability and increased arrhythmia risk (Hinterseer et al., 2010; Sato et al., 2025). Moreover, empagliflozin reduced susceptibility to APD alternans and DADs in both murine and rabbit HFrEF cardiomyocytes (Figure 2). These arrhythmogenic mechanisms were critically dependent on intracellular Ca^2+^-instability in HFrEF cardiomyocytes (Figure 3). However, Na^+^ and Ca^2+^ handling are closely coupled and can form a vicious cycle of arrhythmogenic signaling in HF, involving elevated I_Na,L_, intracellular Na^+^ accumulation, NCX-mediated Ca^2+^ loading, CaMKII activation, and RyR2 dysfunction (Hegyi et al., 2021c). Thus, the improvement of Ca^2+^-dependent electrophysiological abnormalities by empagliflozin may be influenced by altered Na^+^ handling and the resulting secondary effects on intracellular Ca^2+^ homeostasis. The suppression of APD alternans and reduction in STV observed with empagliflozin may also be mediated, at least in part, by its effects on I_Na,L_ and I_K1_, consistent with established roles of these currents in regulating repolarization stability (Zaza et al., 2008; Wagner et al., 2011; Belardinelli et al., 2015; Szentandrassy et al., 2015; Hegyi et al., 2019b; Hegyi et al., 2020b; Hegyi et al., 2026). However, the causal contribution of altered I_Na,L_ and I_K1_ to these effects was not directly tested in the present study. Collectively, these findings implicate empagliflozin in the modulation of cardiomyocyte electrophysiological properties; however, the upstream molecular targets and signaling pathways responsible for these effects remain incompletely understood (Caballero et al., 2026). Multiple mechanisms have been proposed to mediate empagliflozin’s cardioprotective actions, including modulation of Na^+^ and Ca^2+^ homeostasis, reduction of oxidative stress, attenuation of inflammatory pathways, and effects on metabolic and mitochondrial function (Lopaschuk and Verma, 2020; Dyck et al., 2022; Hegyi and Bers, 2023). With respect to empagliflozin’s favorable electrophysiological effects, two putative targets, NHE-1 and I_Na,L_, have been extensively investigated previously, and were studied here in non-diabetic, non-ischemic HFrEF models. Here we discuss our results in the context of these earlier studies and highlight key differences in findings that may be attributable to variations in empagliflozin concentration, treatment duration, and disease models used.

In HF, the intracellular [Na^+^] is significantly elevated, which can disrupt both sarcolemmal and mitochondrial Na^+^/Ca^2+^ exchange and consequently alter cytosolic and mitochondrial Ca^2+^ homeostasis (Despa et al., 2002). These changes can establish a vicious cycle of excessive ROS production and chronic CaMKII activation, leading to electrophysiological, contractile, and metabolic dysfunction (Hegyi et al., 2021c). Both increased I_Na,L_ and upregulation of NHE-1 promote increased Na^+^ influx in HF (Baartscheer et al., 2003; Despa and Bers, 2013; Horvath and Bers, 2014). Indeed, numerous studies have demonstrated that selective inhibition of NHE-1 (Baartscheer et al., 2003; Baartscheer et al., 2008) or I_Na,L_ (Belardinelli et al., 2015; Hegyi et al., 2019b; Horvath et al., 2020) is antiarrhythmic, and they provide significant benefits in preclinical HF models. Nevertheless, it remains unclear whether NHE-1 and I_Na,L_ are direct targets of empagliflozin and whether these pathways represent a dominant mechanism mediating empagliflozin’s antiarrhythmic and functional benefits under physiological conditions (Chung et al., 2021; Murphy and Eisner, 2021; Dyck et al., 2022; Hegyi and Bers, 2023). Importantly, prolonged exposure to protease XIV during enzymatic cardiomyocyte isolation (55 min, but not 35 min) abrogates the effects of empagliflozin on NHE-1, which may contribute to discrepancies in the literature (Chen et al., 2023).

NHE-1 has low activity at physiological pH and accounts for ≤8% of total Na^+^ entry in healthy paced cardiomyocytes, but its activity rises steeply in acidosis (Lazdunski et al., 1985; Leem et al., 1999; Despa and Bers, 2013). In HF myocytes, increased glucose uptake combined with reduced glucose oxidation promotes the conversion of pyruvate to lactate, contributing to intracellular acidosis (Nickel et al., 2013). In HFrEF rabbits, NHE-1 activity was markedly increased at low intracellular pH, and acute cariporide treatment (10 μmol/L, 5 min) in isolated myocytes reduced intracellular [Na^+^] and ameliorated Ca^2+^ handling defects (Baartscheer et al., 2003). Chronic (2 months) *in vivo* cariporide treatment in HFrEF rabbits reduced cardiac hypertrophy, improved contractile function, attenuated APD and QT prolongation, and prevented [Ca^2+^] after-transients following cessation of pacing (Baartscheer et al., 2008). In these rabbits, steady-state plasma concentration of cariporide was ∼5 μmol/L (Baartscheer et al., 2008), roughly 5-fold higher than required for near complete NHE-1 inhibition (IC_50_ = 41 nmol/L) (Kim et al., 2007). Notably, cariporide (1 μmol/L, 2 min) modestly reduced the veratrine-induced I_Na,L_ (by 22%) in rat ventricular myocytes (Chattou et al., 2000). In hiPSC-CMs, cariporide pretreatment (10 μmol/L, 24 h) reduced peak transient (I_Na,T_) without altering I_K1_ (Dago et al., 2022). However, in mouse and rabbit ventricular myocytes, cariporide (1 μmol/L, 3 h) did not affect dV/dt_max_, a surrogate measure of I_Na,T_ function (Figures 1G,H). In our experiments, cariporide pretreatment also failed to attenuate APD prolongation in non-ischemic HFrEF models (Figures 1A–E) and only modestly reduced DADs in TAC but not in HFrEF rabbit cardiomyocytes (Figures 2G,H). The lack of a significant effect of cariporide in our study may reflect the normoxic conditions and relatively physiological intracellular pH maintained during electrophysiological recordings.

### 4.2 Potential mechanisms underlying the effects of empagliflozin on Na^+^ channel function

I_Na,L_ is a small current in healthy cardiomyocytes but is markedly increased in HF, in part via CaMKII-dependent phosphorylation of Na^+^ channels (Wagner et al., 2006; Zaza et al., 2008; Horvath et al., 2013; Glynn et al., 2015; Hegyi et al., 2019b). Several studies have reported effects of empagliflozin on cardiac Na^+^ channels; however, the findings are inconsistent and can be broadly grouped into three mechanistic frameworks, described below.

1. *Direct inhibition of Na* ^*+*^ *channels.* Empagliflozin may directly bind to Na^+^ channels and inhibit pathogenic I_Na,L_. In TAC murine cardiomyocytes, 10 μmol/L empagliflozin acutely inhibited I_Na,L_ without affecting the peak transient Na^+^ current I_Na,T_ (Philippaert et al., 2021). Similarly, in HEK293T cells expressing recombinant human Na_V_1.5 (the predominant cardiac Na^+^ channel isoform), empagliflozin inhibited the H_2_O_2_-induced I_Na,L_ with an IC_50_ of 0.79 μmol/L (Philippaert et al., 2021). In contrast, in murine cardiomyocytes, inhibition of H_2_O_2_-induced I_Na,L_ occurred with a higher empagliflozin IC_50_ of 5.68 μmol/L (Hegyi et al., 2022b). Computational modeling suggested that empagliflozin binds to residues F1760 and W1345 within the domain III-IV linker of Na_V_1.5 (corresponding to the local anesthetic binding site in the inner pore), and mutations at these sites abolished empagliflozin’s inhibitory effect (Philippaert et al., 2021). Because this putative binding site is located deep within the channel pore, access to the site may require higher drug concentrations and/or prolonged exposure, potentially explaining the absence of acute I_Na,L_ inhibition observed in our study. Notably, unlike classical Na^+^ channel inhibitors, empagliflozin did not exhibit frequency-dependent inhibition (Philippaert et al., 2021). Empagliflozin at 10 μmol/L concentration also selectively inhibited I_Na,L_ in long QT syndrome type 3 (LQT3) mutations affecting the inactivation gate, without altering I_Na,T_ or channel kinetics (Lunsonga et al., 2025). In contrast, for mutations within the S4 voltage-sensing region, empagliflozin inhibited both I_Na,T_ and I_Na,L_, altered channel gating, and slowed recovery from inactivation (Lunsonga et al., 2025). Similar mechanisms have been reported for dapagliflozin (another SGLT2 inhibitor); however, dapagliflozin inhibited peak I_Na,T_ only at substantially higher IC_50_ values: 15.16 μmol/L and 29.66 μmol/L in atrial-like and ventricular-like hiPSC-CMs, respectively (Paasche et al., 2024).
2. *Modulation of PTMs.* Empagliflozin may indirectly suppress I_Na,L_ by inhibiting PTMs that enhance I_Na,L_ in HF. Empagliflozin pretreatment (30 min–4 h), but not acute application, reversed I_Na,L_ enhancement in cardiomyocytes from two translational murine models of obese-diabetic HFpEF (Hegyi et al., 2022a; Hegyi et al., 2022b), and in human cardiomyocytes from patients with aortic stenosis and phenotypic features of HFpEF (Mustroph et al., 2022). These effects were also mimicked by selective CaMKII inhibition. Given that I_Na,L_ was measured in the presence of 10 mmol/L EGTA in the pipette solution, a common practice in conventional voltage-clamp experiments, this condition likely underestimates both the HFrEF-associated increase in I_Na,L_ and the rescuing effect of empagliflozin. Moreover, empagliflozin failed to suppress ATX-II- or veratridine-induced I_Na,L_, as well as the enhanced I_Na,L_ in Na_V_1.5 S571E, a phosphomimetic mutant of a key CaMKII phosphorylation site (Glynn et al., 2015), supporting a PTM-dependent mechanism (Mustroph et al., 2022). In line with this interpretation, empagliflozin reduced CaMKII autophosphorylation and Na_V_1.5 phosphorylation in human end-stage HF samples (Mustroph et al., 2022). Additionally, empagliflozin attenuated cardiac inflammation via suppression of nucleotide-binding domain-like receptor protein 3 (NLRP3) activation through a Ca^2+^-dependent mechanism in murine HFrEF hearts (Byrne et al., 2020), and CaMKII has been implicated in NLRP3 activation (Suetomi et al., 2019; Heijman et al., 2020).
3. *Increased Na* ^*+*^ *channel functional expression via pharmacological chaperoning.* In hiPSC-CMs, empagliflozin (1 μmol/L, 24 h) increased peak I_Na,T_ density and induced a leftward shift in steady-state inactivation without affecting activation, thereby narrowing the Na^+^ “window” current that may contribute to reduced I_Na,L_ (Dago et al., 2022). Similarly, empagliflozin (1 μmol/L, 24 h), but not cariporide (10 μmol/L, 24 h), increased I_Na,T_ in dystrophin-deficient *mdx* ventricular myocytes (Sauer et al., 2024). Mechanistically, empagliflozin enhanced anterograde trafficking of Na_V_1.5, acting as a pharmacological chaperone (Sauer et al., 2025). Mexiletine, a Na^+^ channel inhibitor and class 1B antiarrhythmic drug, has likewise been shown to promote Na_V_1.5 trafficking to the plasma membrane. Mutating the local anesthetic binding site (Y1767A) in human Na_V_1.5 abolished the ability of both empagliflozin and mexiletine to increase functional I_Na,T_ expression (Sauer et al., 2025). Empagliflozin also increased dV/dt_max_ in HFrEF cardiomyocytes (Figures 1G,H); however, this effect may be, in part, secondary to normalization of RMP (and thus increased Na^+^ channel availability) or mediated by PTMs (e.g., CaMKII-dependent phosphorylation) rather than a direct effect on Na^+^ channels. Further studies are needed to determine whether empagliflozin alters Na^+^ channel trafficking or I_Na,T_ in HFrEF.

### 4.3 Antiarrhythmic effects of empagliflozin beyond Na^+^ homeostasis

Beyond Na^+^ fluxes, empagliflozin has also been reported to affect K^+^ currents. Empagliflozin (1 μmol/L, 24 h) increased inward I_K1_ density (at −120 mV) in hiPSC-CMs (Dago et al., 2022) consistent with our findings in HFrEF cardiomyocytes (Figures 4E–H) using a shorter cell incubation time (1 μmol/L, 3 h). Interestingly, empagliflozin increased the physiologically relevant outward I_K1_ to a greater extent than inward I_K1_ in HFrEF murine cardiomyocytes (Figure 4G), suggesting potential alterations in the expression of K_ir_2. x channel subtypes and/or key gating modulators, such as Mg^2+^ and positively charged polyamines, that govern channel rectification. Other key ionic currents altered in HFrEF, including I_Ca,L_, I_NCX_, I_Kr_, and I_Ks_, may also be modulated by empagliflozin and could contribute to the normalization of APD; however, this possibility remains to be investigated (Hegyi et al., 2018a; Hegyi et al., 2018b; Hegyi et al., 2019b). Notably, empagliflozin (5 μmol/L, 2 h) modestly increased I_Kr_ and I_Ks_ in zebrafish hearts (Karpushev et al., 2022); however, whether similar effects occur in human or larger animal hearts, in which these currents are critical determinants of cardiac repolarization, remains unknown.

Empagliflozin can also provide additional cardioprotective effects through actions on non-cardiomyocytes. These include improved nitric oxide (NO) signaling and vascular tone, associated with reduced inflammation and ROS in coronary endothelial cells (Wang et al., 2025), attenuation of endothelial to mesenchymal transition and fibroblast activation (Schmidt et al., 2024), and suppression of myofibroblast differentiation (Baufays et al., 2025). Together, these mechanisms may limit cardiac fibrosis and provide beneficial paracrine effects on cardiomyocytes, thereby further reducing arrhythmia susceptibility in HF.

### 4.4 Conclusions and perspectives

Overall, our findings in murine and rabbit HFrEF cardiomyocytes are most consistent with mechanisms *(2)* and *(3)* described above, supporting the hypothesis that empagliflozin may either reverse PTM-dependent I_Na,L_ enhancement and ameliorate the reduced I_K1_ and potentially I_Na,T_, and/or act as a pharmacological chaperone. Distinguishing between these possibilities will require further investigation. Previous studies have also shown that supratherapeutic cariporide and empagliflozin concentrations (∼10 μmol/L) can directly inhibit Na_V_1.5, highlighting the potential for off-target effects that should be considered when interpreting their actions in preclinical studies.

In summary, empagliflozin exerted complex antiarrhythmic effects directly in HFrEF cardiomyocytes that extend beyond acute inhibition of NHE-1 and may involve modulation of cellular signaling pathways and PTMs affecting multiple ion channels, as well as improved intracellular Na^+^ and Ca^2+^ handling. These effects may enhance electrical stability and contribute to attenuation of APD prolongation and reduction of cellular proarrhythmic mechanisms, including STV, APD alternans, and DADs. Although the precise molecular target(s) of empagliflozin in cardiomyocytes remains unclear, our data indicate that empagliflozin has antiarrhythmic properties associated with improved electrical stability in failing cardiomyocytes.

## 5 Limitations

Several limitations should be noted. First, only male animals were studied; thus, further studies are required to determine whether empagliflozin responses are sex-dependent. Second, this study was performed in a non-ischemic HFrEF model, and the generalizability of empagliflozin’s effects to ischemic HFrEF remains to be determined. Third, our experiments focused on cellular mechanisms of arrhythmogenesis. Although the findings suggest that empagliflozin exerts antiarrhythmic effects through favorable modulation of cardiac electrophysiology, these effects should be further evaluated at the tissue and organ levels using whole-heart optical mapping and *in vivo* arrhythmia testing. Fourth, the precise molecular mechanism(s) and direct target(s) of empagliflozin, as well as whether these effects are specific to empagliflozin or represent a broader class effect, remain to be elucidated.

## Data Availability

The data presented in this study are deposited in Dryad at https://doi.org/10.5061/dryad.hmgqnkb06.
